# Enhancing Access to Neuraxial Ultrasound Phantoms for Medical Education of Pediatric Anesthesia Trainees: Tutorial

**DOI:** 10.2196/63682

**Published:** 2025-05-12

**Authors:** Leah Webb, Melissa Masaracchia, Kim Strupp

**Affiliations:** 1 Division of Pediatric Anesthesiology, Department of Anesthesiology, Children's Hospital Colorado, University of Colorado, Denver, CO, United States, 1 7207774579; 2 Northwell, Division of Pediatric Anesthesiology, Department of Anesthesiology, Cohen Children's Medical Center, Zucker School of Medicine at Hofstra/Northwell, New Hyde Park, NY, United States

**Keywords:** anesthesiology, pediatric, ultrasound, education, neuraxial ultrasound, medical education, pediatric anesthesia trainees, anesthesia, trainees, ultrasound-guided, neuraxial techniques, pediatric patients, efficiency

## Abstract

Opportunities to learn ultrasound-guided/assisted (USGA) neuraxial techniques for pediatric patients are limited, given the inherent high stakes and small margin of error in this population. Simulation is especially valuable in pediatrics because it enhances competency and efficiency, without added risk, when learning new skills, specifically those seen with ultrasound-guided regional anesthetic techniques. However, access to simulation opportunities involving the use of phantom models in medical education is limited due to excessive costs. We describe a process for producing ultrasound phantoms by using synthetic ballistic gelatin; these ultrasound phantoms can be used for simulation and are affordable, reproducible, and indefinitely shelf stable. The ultrasound images produced by these phantoms are comparable to those obtained from a real pediatric patient, including the sacral anatomy necessary for caudal epidural blocks, as validated by practicing pediatric anesthesiologists. Phantom models offer a more cost-effective alternative to commercially prepared phantoms, thereby expanding access to realistic simulations for neuraxial ultrasound in pediatric medical education, without the prohibitively high expense.

## Introduction

Opportunities to learn ultrasound-guided/assisted (USGA) neuraxial techniques for pediatric patients are limited, given the inherent high stakes and small margin of error in this population. Simulation is a valuable and effective method for learners—whether used by trainees or experienced clinicians—to enhance their competency, efficiency, and confidence in performing regional anesthetic and neuraxial techniques [1-4]. Ultrasound enhances safety, decreases complications, and improves the efficacy and accuracy of neuraxial blockade in pediatric patients from preterm to adolescence [5-12]. The utility of ultrasound is even more apparent in syndromic children with unusual anatomy, patients who comprise a large subset of the pediatric population that presents for surgery at a young age [13]. Honing pediatric patient–related ultrasound skills in a simulation setting is an ideal scenario for learning without risk. Unfortunately, educational curricula and teaching models lag behind recent advancements in simulation.

Despite efforts to create affordable and reproducible ultrasound phantoms, many lack a realistic appearance, and most are not indefinitely, if at all, shelf stable or portable because they are made of water, agar, gelatin, or other substances or are derived porcine models [3,4,14,15]. The cost of manufactured models that offer all these features can be prohibitively expensive, amounting to several thousand dollars, and these models may generate an inferior simulation experience [14]. Limited access to high-fidelity ultrasound phantoms significantly restricts opportunities for learners to take advantage of low-stakes simulation training and necessitates practicing on live patients, including infants and children, to learn valuable skills—a method with varying degrees of success and much higher stakes. There is a dearth of literature describing spine phantoms that are made with the necessary anatomy to teach pediatric trainees how to use ultrasound to approach the caudal epidural space. We describe a method for creating a realistic, affordable, reproducible, and shelf-stable spine phantom model that allows for the demonstration of key ultrasound images of the spine and caudal anatomy that are required to perform USGA neuraxial techniques on pediatric patients. Furthermore, the synthetic ballistic gelatin used to produce the phantom model can be reclaimed and reused to make “fresh” models for an indefinite period of time, allowing for multiple practice sessions without additional costs.

## Methods

### Overview

We present a tutorial describing the construction of an ultrasound phantom of the spine, based on similar previous descriptions [16]. Notably however, our model includes both lumbar anatomy and sacral anatomy, which are lacking in previously published iterations but are essential for learning pediatric-specific neuraxial sonoanatomy. Additionally, we completely submerged our spine model in ballistics gel, creating stable, flat surfaces surrounding the spine to facilitate scanning the model in multiple orientations, which simulates the use of ultrasound for prone, lateral, and sitting positions. Further, an anonymous survey was sent to 10 practicing attending pediatric anesthesiologists to evaluate the similarity between the ultrasound images generated from the phantom and images from a real patient. Three ultrasound views were evaluated for likeness and accuracy on a 5-point Likert scale.

### How to Create a Phantom Model

The following stepwise process can be used to create a spine phantom:

- Step 1: Preheat a portable oven to 250-270 °F (121-132 °C). A portable oven is preferable, as it can be used outside to prevent inhalation of the unpleasant smell from melting gel. It is critical to review the manufacturer’s guidelines; ensure that the ballistics gel is always melted in well-ventilated areas; and ensure that caution is used to avoid overheating the gel, as it could light on fire.
- Step 2: Cut or tear ballistics gel into smaller pieces for melting.
- Step 3: Place the gel into an oven-safe pan (either a mold pan or an extra container), with the goal of melting the gel to create a 1- to 3-inch gel layer at the bottom of the pan.
  - This layer mimics the soft tissue covering the spinous processes. Add more gel to create a thicker layer, if desiring to create a model with greater depth to the epidural space.
  - Generally, it is preferable to melt the gel in an extra oven-safe container and pour it into a mold pan for each subsequent step; however, for the first layer, the gel can preferentially be melted directly in the mold pan.
- Step 4: Melt the gel in the oven until all bubbles are gone. This step takes about 2 to 4 hours, depending upon the amount of gel melted. It is critical to minimize bubbles in this layer, as this is the surface that will be scanned with ultrasound.
- Step 5: Allow the bubble-free layer to cool significantly (approximately 30-60 min). Then, place the spine model into the pan, with spinous processes facing down toward the bottom of pan and touching the gel, and press it very gently into the gel. Hold or secure the spine model in place until the gel sets and the model is not moving in the pan (10-20 min).
  - During the first cooling period, the gel should be cool enough to touch and be starting to firm up, with some resistance to pressure from a fingertip, but it should be soft enough to envelop the tips of the spine model’s spinous processes.
  - If free-pouring gel into a mold pan (rather than melting gel directly in it, as is preferable), aim to pour toward one side/corner of the pan to minimize air bubbles. A ladle can be used to pour gel into the mold pan, again aiming to pour into one side/corner of the pan (rather than fanning) to minimize bubbles.
  - If many large bubbles are present, consider placing the mold pan back into the oven and cooking further until bubbles are gone (a few small bubbles are generally not problematic).
  - Hot gel will soften the spine model and result in curved spinous processes. Therefore, press only hard enough on the model to make slight contact between the tips of spinous processes and the gel; this contact secures the model to hold it in place in future steps.
- Step 6: Allow gel in mold pan (containing 1- to 3-inch gel layer) and spine model to cool completely.
- Step 7: Once cool, pour more melted gel (see step 3 for melting instructions) into the mold pan until the spine model is completely covered.
  - Pour quickly and to one corner or side at the coccyx-end of the model.
  - Bubbles in this step are not as concerning because this surface will be placed facing down and will not be scanned.
  - Bubbles will continue to rise to the top for several minutes as the gel cools; large bubbles can be popped/opened to create a flatter surface on this side of the phantom, though it is not necessary to do so.
- Step 8: Allow the mold pan (which should now contain the gel-covered spine model) to cool completely, preferably overnight, until the gel is solid.
- Step 9: Remove phantom from pan, using firm but gentle traction on the gel.
  - It may help to run an offset spatula (or another flat, thin tool, such as a butter knife) along the edges of the phantom and pan to help separate the phantom from the mold pan.
  - Once loosened, it can be helpful to stand the pan upright on the short side and slide fingers between the gel and pan as deep as possible to fully free the top side of the gel. Then, firmly push down, while continuously pulling out, on the gel until it releases from the pan.
- Step 10: Store phantom at room temperature, with spinous process side up. To clean, use water and a lint-free towel.

**Figure 1.**
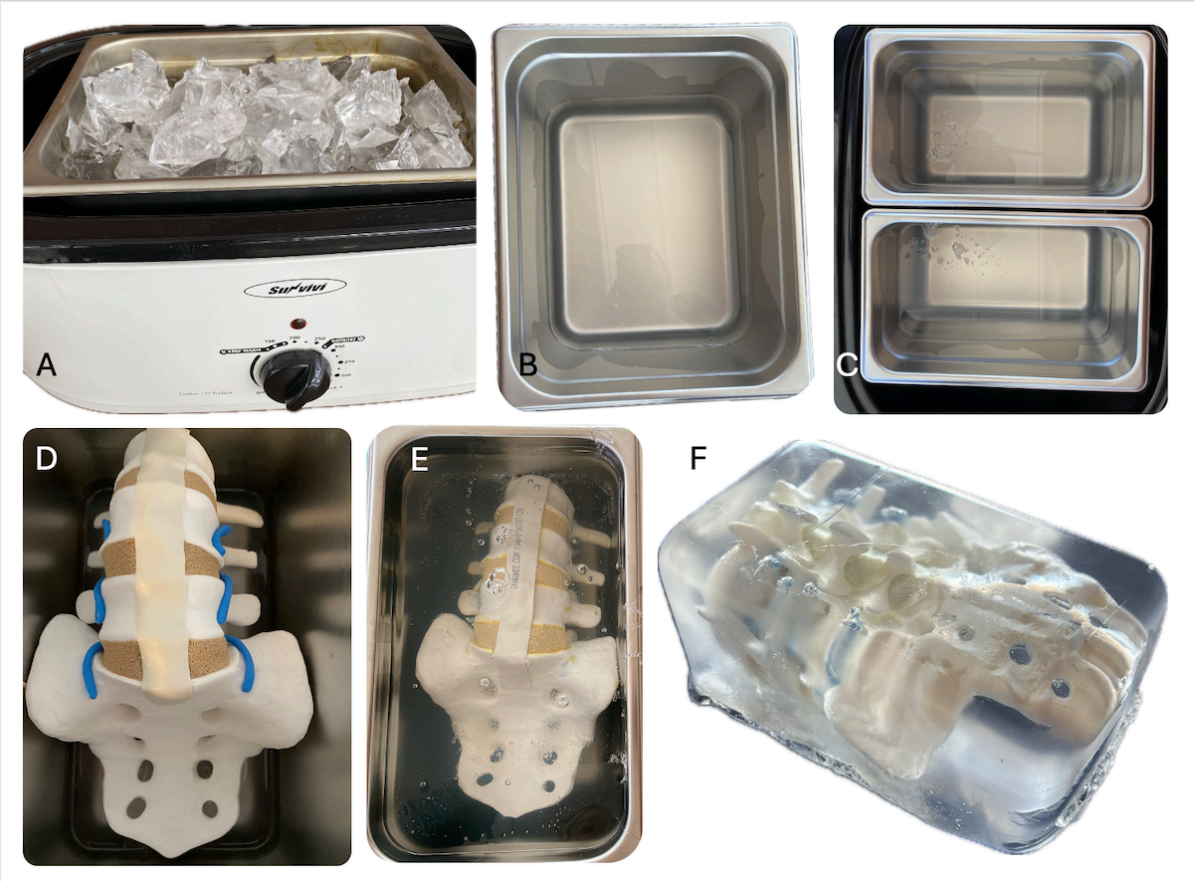
Stages of phantom production, with A to C showing steps 1 to 4, D showing steps 5 and 6, E showing steps 7 and 8, and F showing step 9. (A): Cut gel in an extra container placed inside a portable oven set to 270 °F (132 °C). (B): Melted, bubble-free gel in the extra container. (C): Two mold pans. (D): Spine model, with anterior side up, placed in cooled, bubble-free layer. (E): Spine model submerged completely in gel and cooled. (F): Completed spine phantom that has cooled completely and has been removed from the mold pan.

Figure 1 shows correlated pictures of the stepwise process and final phantom model.

### Materials

Multiple options exist for the materials that are used to create a phantom model for practicing neuraxial ultrasound skills. Table 1 outlines those used by the authors, along with purchase sites and prices. Each phantom is composed of a spine model embedded in ballistics gel. Additional necessary items are reusable for multiple production cycles. Supplies include an oven that can sustain 250-270 °F (121-132 °C; US $119 for a portable oven), an oven-safe mold (US $9), and an extra oven-safe container (US $11). Optional items include a ladle or another heatproof tool for scooping melted gel. The ladle can be useful for more precision in transferring the gel into the pan. It does potentially create more bubbles than pouring directly; however, bubbles are mitigated by placing the pan back into the oven. The ladle is also useful for ensuring that the anterior side of the model is completely covered with gel and that any bubbles remaining on the anterior side do not interfere with ultrasound scanning. Furthermore, because the spine model is completely submerged in ballistics gel, an offset spatula may be helpful for loosening and releasing the phantom from its mold.

**Table 1.** List of materials, where to purchase, costs, and notes on pertinent information.

Item and description		Purchase site	Cost	Notes
Oven (portable)				
	“Sunvivi 22-Quart Roaster Oven”	Amazon.com (ASIN^a^: B07K25WBZ4)	US $119	Any oven that can sustain 250‐270 °F (121-132 °C).
Ballistics gel				
	“10% FBI Gel Block”	Clearballistics.com (SKU^b^: 852844007000)	US $76 + shipping	Makes ≥4 phantoms.
Spine model				
	“Spine, Lumbar Vertebrae with Nerve Roots and Ligamenta Flava, L3-Sacrum, Solid Foam”	Sawbones.com (SKU: 1340-1)	US $161 + shipping	Preferred.
	“Medical Human Lumbar Spine Demonstration Model Anatomical Model Lumbar Vertebrae Sacrum & Coccyx, with Herniation Disc,for Science Classroom Study Display Teaching Medical Model 15 Inch Hight”	Amazon.com (ASIN: B074JCS4SC)	US $34	Less expensive. Requires removal of some vertebrae to fit recommended oven-safe mold. Alternative option is 3D printed model.
Oven-safe mold				
	“1/4 size 6” Deep Steam Table Pan”	Webstaurantstore.com (item number: 4070469	US $9	Any oven-safe receptacle that is similar in size to spine model. Can be purchased from local restaurant supply store.
Extra oven-safe container				
	“1/2 Size 6” Deep Steam Table Pan”	Webstaurantstore.com (item number: 4070269)	US $11	Used to melt bigger volume of gel.
Gel dye				
“Tone dye”	Humimic Medical [17]	US $35	Used to opacify gel; comes in a variety of skin tone colors.

a Amazon Standard Identification Number.

b Stock keeping unit.

### Cost

The cost for our preferred spine model is approximately US $161, but a more cost-effective version with fewer vertebrae can be purchased for US $34. The more expensive model is preferred due to the ease of placement in the mold, image quality on ultrasound scans, and representation of more neuraxial structures (spinal nerves and ligamentum flavum). Newer 3D printing technology allows for the printing of customizable and cost-effective pediatric spine models that could alternatively be used in our phantom. Ballistics gel priced at US $76 allows for the production of 4 or more phantoms. The additional items previously mentioned can amount to a cost between US $139 and US $150.

## Results

**Figure 2.**
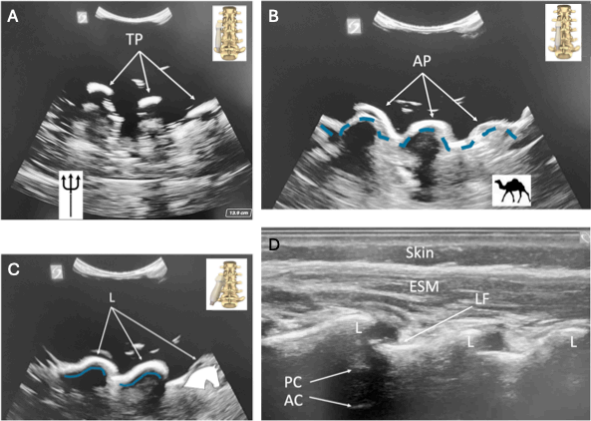
Parasagittal images from phantom (**A, B, and C**), with probe placement relevant to bony anatomy and ultrasound indicator oriented cephalad, and patient (**D**), with ultrasound indicator oriented caudad. (A): Parasagittal TP view (“trident” sign). (B): Parasagittal AP view (“camel hump” sign; dashed blue line shows “camel hump” outline). (C): Parasagittal oblique interlaminar view (“horse head” or “sawtooth” sign; blue line shows “horse head” outline) in phantom. (D): Parasagittal oblique interlaminar view in patient. AC: anterior complex (interface of anterior dura and vertebral body); AP: articular process; ESM: erector spinae muscle; L: lamina; LF: ligamentum flavum; PC: posterior complex (interface of ligamentum flavum and posterior dura); TP: transverse process.

**Figure 3.**
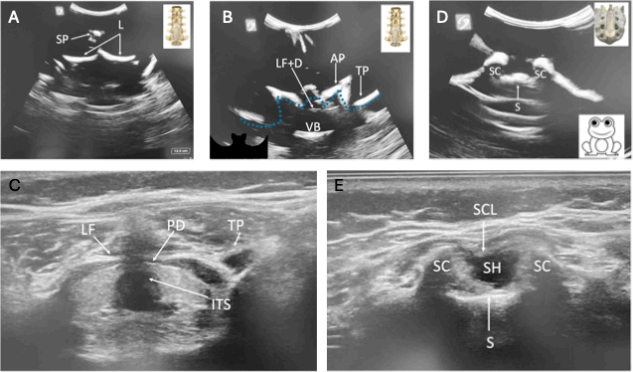
Transverse images from phantom (**A, B, and D**), with probe placement relevant to bony anatomy, and patient (**C and E**); ultrasound probe indicator is oriented left in all images. (A): Transverse midline SP view from phantom. (B): Transverse interspinous (interlaminar) view (“bat” or “bat wing” sign; dotted line shows “bat wing” outline) from phantom. (C) Transverse interspinous (interlaminar) view from patient. (D): Transverse SC view (“frog” or “frog eye” sign) from phantom. (E): Transverse SC view from patient. AP: articular process; ITS: intrathecal space; L: lamina; LF: ligamentum flavum; LF+D: ligamentum flavum+dura (ie, posterior complex); PD: posterior dura; S: sacrum; SC: sacral cornua; SCL: sacrococcygeal ligament; SH: sacral hiatus; SP: spinous process; TP: transverse process; VB: vertebral body.

There are 6 views that are critical to performing USGA neuraxial procedures; each is easily obtained from the ultrasound phantom:

- Parasagittal views (Figure 2): transverse process (“trident” sign), articular process (“camel hump” sign), and oblique interlaminar (“horse head” or “sawtooth” sign) views
- Transverse midline views (Figure 3): spinous process, interspinous process/interlaminar (“bat” or “flying bat” sign), and sacral cornua (“frog” or “frog eye” sign) views

Each of these views is demonstrated in Figures2  3, with an additional image of the ultrasound probe placement in relation to the bony landmarks of the lumbosacral spine and patient sonoanatomy, where available. Several spine images from a 6-month-old male infant (written consent obtained from parent) are shown (Figures2D, 3C,  3E) beside the phantom images for comparison. Because the spine model lacks certain elements, not all structures appear on the phantom scan, and some cannot be obtained.

Phantom images were evaluated for likeness and accuracy via comparison to actual patient images by 10 practicing attending anesthesiologists. Each image was graded on a 5-point Likert scale for how similar it appeared to the actual patient image. All 10 respondents agreed or strongly agreed that the transverse sacral cornua view (“frog” sign) and parasagittal oblique interlaminar view (“horse head” sign) were similar to those of real patients. Of the 10 respondents, 8 agreed or strongly agreed that the transverse interspinous view (“bat wing” sign) was similar between the phantom and real patient images, 1 respondent was neutral, and 1 respondent somewhat disagreed.

## Discussion

Unlike previous phantoms described by Morrow et al [16], Mashari et al [14], and others, our spine phantom generates ultrasound images and views that closely replicate the sonoanatomy of a pediatric patient (Figures2D, 3C,  3E). A key advancement in our design is the incorporation of the sacrum and sacral hiatus—critical structures needed for visualizing the caudal space, which is a technique that is often used in accessing the neuraxis in pediatric patients. Furthermore, by fully submersing the spine model in ballistics gel, our phantom offers superior stability during scanning and allows for repositioning to simulate sitting, lateral, and prone patient orientations. The enhanced design ensures a more realistic training experience, thereby helping practitioners develop the precise skills necessary for pediatric neuraxial techniques.

Practicing pediatric anesthesiologists overall found our phantom’s ultrasound images comparable to ultrasound images of real pediatric anatomy, particularly for the transverse sacral cornua (“frog” sign) and parasagittal oblique interlaminar (“horse head” sign) views. However, while responses for the transverse interspinous (interlaminar) view (“bat wing” sign) were generally positive, some noted minor discrepancies between the phantom images and those of real patients. Given that the phantom lacked several ligaments and the spinal canal seen in real patients, this feedback provides an opportunity for improvement in future phantom models, which could be addressed by the techniques described by Morrow et al [16] (spinal canal) and Mashari et al [14] (ligaments).

Ultrasound has been used to identify anatomical landmarks for epidural or spinal neuraxial procedures and to identify placement of catheters that are inserted in the caudal space and threaded to the lumbar or thoracic space in pediatric patients [5,6,9-12]. The creation of ultrasound phantoms, as described in this paper, can increase access to ultrasound simulation and enhance opportunities for learning critical procedural skills in a low-stakes environment [1-4,14,16]. To meet these needs, we created a phantom that is indefinitely shelf stable, reproducible, and cost-effective (approximately US $92 to US $219 per phantom, including the materials listed plus the reusable materials). By modifying the previous technique described by Morrow et al [16], our phantom was specifically designed to image the sacral cornua and to easily scan in the prone or lateral positions, which are essential features for training anesthesia clinicians in pediatric neuraxial sonoanatomy.

The use of spine phantoms was previously limited by their costs; however, budget-friendly spine phantoms created with readily available materials produce a realistic feel when palpating for anatomic landmarks [14] and generate many of the views required to perform neuraxial USGA procedures [14,16]. These phantoms also replicate sonoanatomy with high fidelity, as demonstrated by Mashari et al [14], who actually found that their low-cost model resulted in superior fidelity for ultrasound imaging when compared to an expensive, commercially available task trainer.

There are some limitations to the phantom described herein, of which many can be attributed to the absence of more complex anatomical structures. Although some views and sonoanatomy cannot be identified without these structures, easy solutions are available if needed. For example, our model has a fused sacrum, as is common in adults; therefore, scanning of the sacrum in the sagittal plane—a useful technique for performing in-plane USGA caudal epidural blocks in infants and children—is futile. This problem can be relieved by obtaining an anatomically correct spine model that is reflective of infants or young children, either through purchase or through 3D printing [14,18]. Models of pediatric spines with both normal anatomy and abnormal anatomy could be made via 3D printing, enhancing the pediatric-specific simulation experience; however, access can be limited and may be costly when considering the initial monetary investment in a 3D printer.

The phantom described also lacks contents of the spinal canal, rendering it inadequate for simulating access to the intrathecal space for spinal blockade. Inserting fluid-filled tubing into the empty spinal canal (a technique described by Morrow et al [16]) prior to pouring melted gel on the model could provide a potential solution. However, while this added feature can present itself as another useful learning tool, we found that needling the phantom degrades the image quality over time and should be considered when deciding whether to include a spinal canal in future models. Other potential options for making a more complete model include adding a ligamentum flavum by using silicone paste [14]. This technique may be useful for creating a sacrococcygeal ligament, which is an important landmark when performing USGA caudal blocks while using the transverse sacral cornua view (“frog” sign).

Of further note, we chose to use clear ballistics gel for our phantoms, since it allows for the direct visualization of spine model structures, which can be very helpful for early learners but is not realistic or comparable to scanning live patients. Products used to opacify the gel can be purchased on the internet (Table 1) if a more realistic option is desired.

Future directions for the use of our spine phantom model center on teaching critical skills and assessing knowledge of and comfort with high-stakes procedures in novice trainees. Further evaluation of our phantom should focus on the effectiveness of the phantom as a teaching tool.

By constructing a reproducible, affordable, and shelf-stable spine phantom that can be scanned to generate images and sonoanatomy of the infant and child neuraxis, trainees can be provided with a low-stakes environment in which they can learn how to perform high-stakes regional anesthesia blocks. By addressing the limitations of previous models, our phantom provides an affordable, high-fidelity tool that enhances access to realistic neuraxial ultrasound training for pediatric trainees.

## Abbreviations

USGA: ultrasound-guided/assisted
